# 2-Oxo-2*H*-chromen-7-yl *tert*-butyl­acetate

**DOI:** 10.1107/S2414314625001890

**Published:** 2025-03-04

**Authors:** Hypolite Bazié, Eric Ziki, Sorgho Brahima, Veroarisinima Ratsimbazafy, Patrick Roge, Emmanuel Wenger, Abdoulaye Djandé, Claude Lecomte

**Affiliations:** a Laboratory of Molecular Chemistry and Materials (LC2M), University Joseph KI-ZERBO, 03 BP 7021 Ouagadougou 03, Burkina Faso; b Laboratory of Matter Environmental and Solar Energy Sciences Research Team: Crystallography and Molecular Physics University Félix Houphouët-Boigny 08 BP 582 Abidjan 22 Côte d’Ivoire; c Laboratory of Solid State Physics and Experimental Physics, University of Antananarivo, BP 566, Antananarivo 101, Madagascar; d CRM2, CNRS-Université de Lorraine, Vandoeuvre-lès-Nancy CEDEX BP 70239, France; University of Aberdeen, United Kingdom

**Keywords:** crystal structure, Hirshfeld surface, coumarin, hydrogen bond

## Abstract

In the title compound, C_15_H_16_O_4_, the dihedral angle between the 2*H*-chromen-2-one ring system and the *tert*-butyl­acetate moiety is 72.72 (9)°. In the crystal, the mol­ecules are connected through C—H⋯O hydrogen bonds, generating *C*(6) chains and *R*_2_^2^(20) loops that are reinforced by weak aromatic π–π stacking inter­actions. The H⋯H, H⋯O/O⋯H, H⋯C/C⋯H and C⋯C contacts contribute 50.6, 29.1, 8.5 and 6.8%, respectively, to the Hirshfeld surface.

## Abstract 2

In the title compound, the dihedral angle between the 2*H*-chromen-2-one ring system and the *tert*-butyl­acetate moiety is 72.72 (9)°. In the crystal, the mol­ecules are connected through C—H⋯O hydrogen bonds, generating *C*(6) chains and *R*_2_^2^(20) loops that are reinforced by weak aromatic π–π stacking inter­actions.

## Graphical abstract

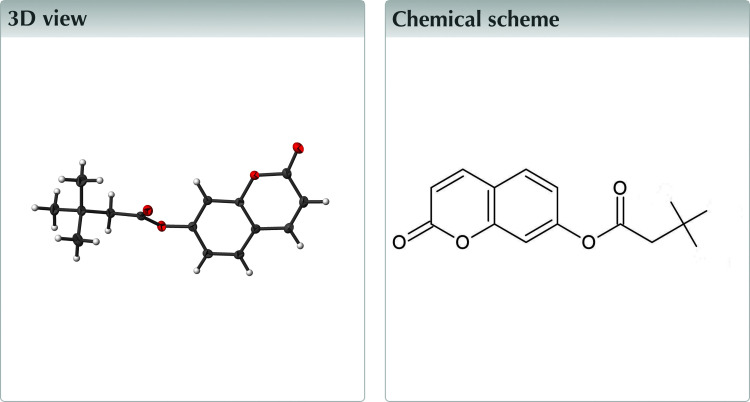

## Structure description

The title coumarin derivative, C_15_H_16_O_4_ (**I**), was synthesized by a research team led by Professor Djandé (LC2M, Ouagadougou, Burkina Faso) as part of the AFRAMED project (Kenfack Tsobnang *et al.*, 2024 ▸). Coumarin-derived compounds exhibit various biological activities, such as anti­cancer (Yadav *et al.*, 2024 ▸; Rawat *et al.*, 2022 ▸), anti­coagulant (Singh *et al.*, 2019), anti-inflammatory (Todeschini *et al.*, 1998 ▸) and anti-glaucoma (Ziki *et al.*, 2023 ▸) properties.

**Figure 1:**
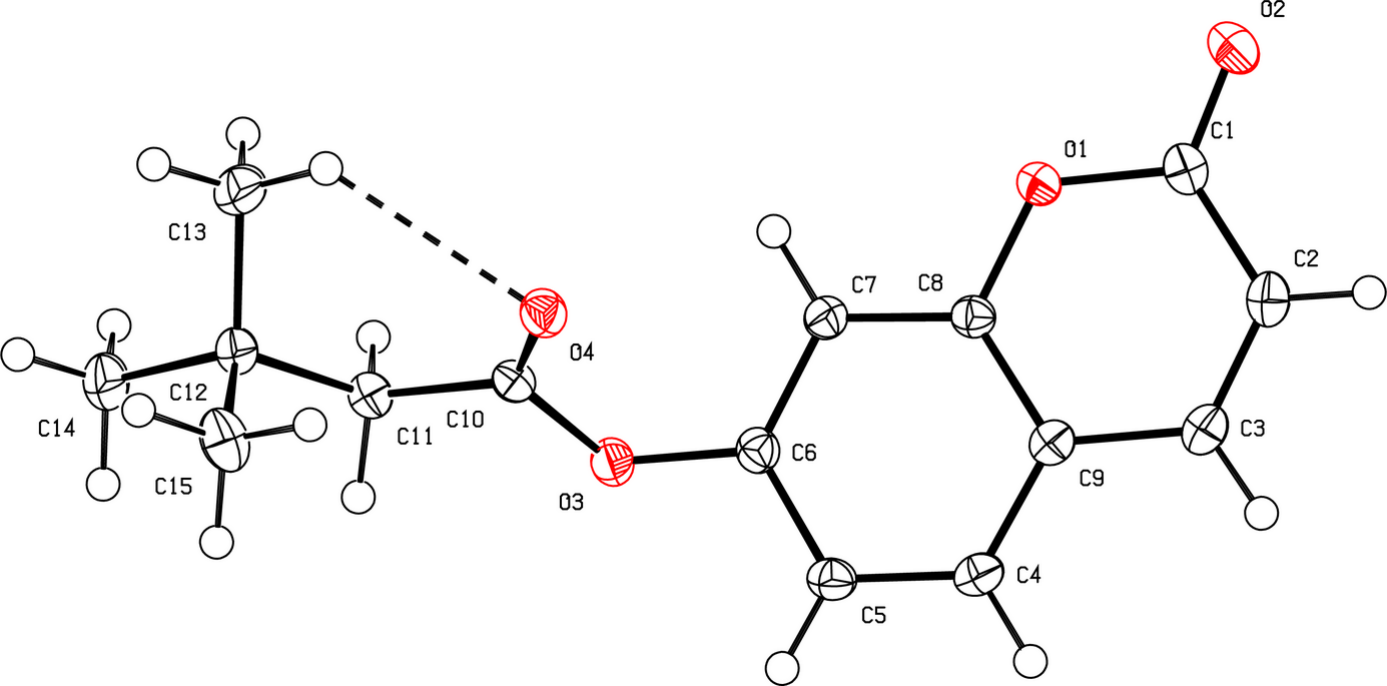
The mol­ecular structure of (**I**) with displacement ellipsoids drawn at the 50% probability level.

As shown in Fig. 1 ▸, the 2*H*-chromen-2-one moiety formed by atoms C1–C9/O1/O2 in (**I**) is almost planar with an r.m.s deviation of 0.027 Å and the dihedral angle between this ring system and the plane formed by atoms C10–C12/C14 in the *tert*-butyl­acetate moiety is 72.72 (9)°. An *S*(6) ring motif resulting from an intra­molecular C13—H13*B*⋯O4 hydrogen bond is observed (Table 1 ▸). The plane passing through atoms C10–C12/C14 of the *tert*-butyl­acetate moiety contains the ester function atoms (r.m.s = 0.228 Å), but methyl atoms C13 and C15 atoms are on either side of this plane with deviations of 1.275 (1) and −1.244 (1) Å, respectively.

**Figure 2:**
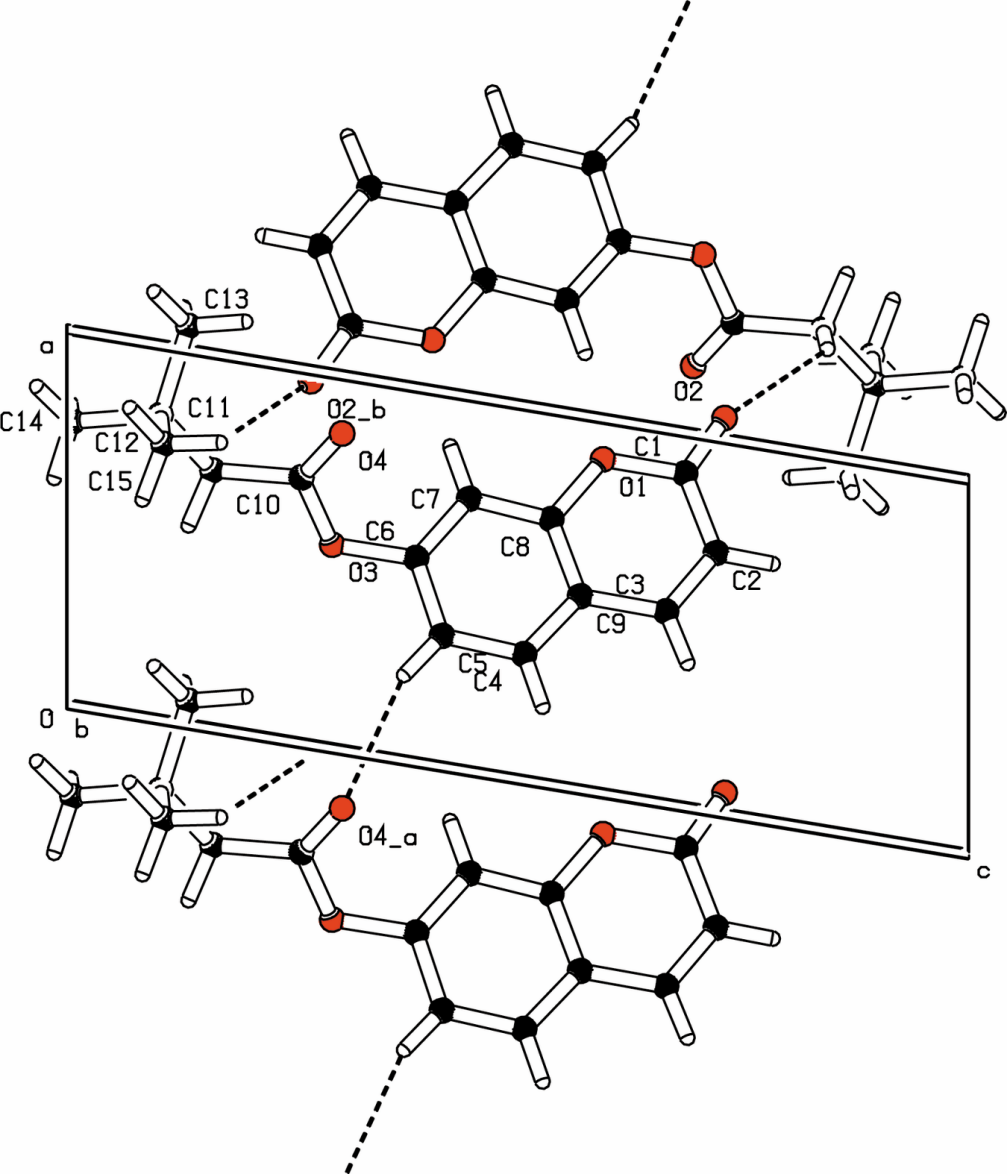
Part of the crystal of (**I**) showing the formation of an undulating network along the *b* axis [*C*(6) and 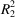
(20) motifs]. Dashed lines indicate hydrogen bonds.

**Figure 3:**
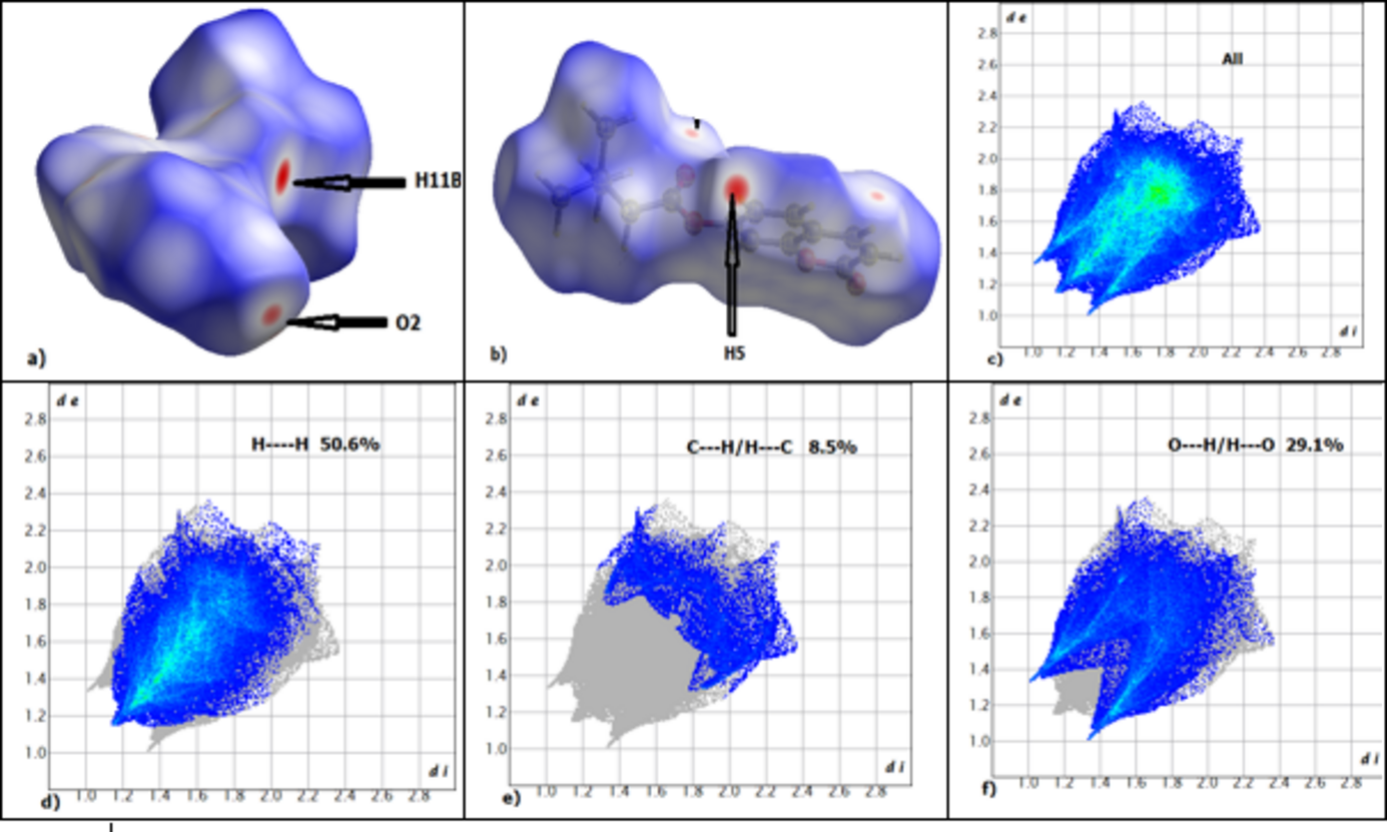
(*a*), (*b*) Hirshfeld surface of (**I**) mapped over *d*_norm_, (*c*) overall two-dimensional fingerprint plot of and those delineated into contributions from different contacts: (*d*) H⋯H, (*d*) H⋯C/C⋯H and (*e*) H⋯O/O⋯H.

In the crystal of (**I**), mol­ecules are linked by weak hydrogen bonds of the C—H⋯O type. A pair of C11—H11*B*⋯O2(−*x* + 2, −*y*, −*z* + 1) hydrogen bonds generates a centrosymmetric 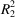
(20) loop, as shown in Fig. 3. The C5—H5⋯O4(*x * − 1, *y*, *z*) hydrogen bonds form *C*(6) chains propagating in the [100] direction (Fig. 2 ▸). Aromatic π–π stacking inter­actions between the pyrone ring (centroid *Cg*1) and benzene ring (centroid *Cg*2) of a symmetry-related (1 − *x*, −*y*, 1 − *z*) mol­ecule reinforce the cohesion of mol­ecules [*Cg*1⋯*Cg*2 = 3.5485 (8) with a slippage of 1.042 Å],

The Hirshfeld surface and two-dimensional fingerprint (FP) plot of **(1**) (Fig. 3 ▸) generated by *CrystalExplorer21.5* (Spackman *et al.*, 2021 ▸) confirmed the above inter­actions. The fingerprint plots show the different contributions of the atoms in the crystal-to-surface contacts. The most important contributions are H⋯H and H⋯O/O⋯H contacts with 50.6 and 29.1%, respectively (Fig. 3 ▸*d* and 3*f*). The H⋯C/C⋯H and C⋯C contacts contribute 8.5 and 6.8%, respectively. These values are close to those of 2-oxo-2*H*-chromen-6-yl 4- *tert*-butyl­benzoate (Kenfack Tsobnang *et al.*, 2024 ▸).

## Synthesis and crystallization

To a solution of *tert*-butyl­acetyl chloride (6.2 mmol, 0.9 ml) in dried diethyl ether (16 ml) was added dried pyridine (4.7 molar equivalents; 2.31 ml) and 7-hy­droxy­coumarin (6.17 mmol, 1.00 g) in small portions over 30 min. The mixture was left under agitation at room temperature for 3 h and then poured into 40 ml of chloro­form. The solution was acidified with dilute hydro­chloric acid (5%) until the pH was 2–3. The organic layer was extracted, washed four times with 25 ml of water to neutrality, dried over MgSO_4_ and the solvent removed. The resulting crude product was filtered off with suction, washed with petroleum ether and recrystallized from acetone solution as colorless crystals of the title compound. Yield = 79%, m.p. = 368–371 K.

## Refinement

Crystal data, data collection and structure refinement details are summarized in Table 2 ▸.

## Supplementary Material

Crystal structure: contains datablock(s) I. DOI: 10.1107/S2414314625001890/hb4507sup1.cif

Structure factors: contains datablock(s) I. DOI: 10.1107/S2414314625001890/hb4507Isup2.hkl

Supporting information file. DOI: 10.1107/S2414314625001890/hb4507Isup3.cml

CCDC reference: 2427772

Additional supporting information:  crystallographic information; 3D view; checkCIF report

## Figures and Tables

**Table 1:** Hydrogen-bond geometry (Å, °)

*D*—H⋯*A*	*D*—H	H⋯*A*	*D*⋯*A*	*D*—H⋯*A*
C5—H5⋯O4^i^	0.95	2.50	3.4144 (13)	161
C11—H11*B*⋯O2^ii^	0.99	2.52	3.2523 (13)	131
C13—H13*B*⋯O4	0.98	2.43	3.0924 (14)	124

Symmetry codes: (i) 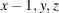; (ii) 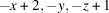.

**Table 2:** Experimental details

Crystal data	
Chemical formula	C_15_H_16_O_4_
*M*_r_	260.28
Crystal system, space group	Triclinic, *P* 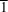
Temperature (K)	100
*a*, *b*, *c* (Å)	6.1599 (9), 7.2029 (11), 15.202 (2)
α, β, γ (°)	98.765 (5), 99.335 (5), 91.228 (5)
*V* (Å^3^)	657.05 (17)
*Z*	2
Radiation type	Mo *K*α
μ (mm^−1^)	0.10
Crystal size (mm)	0.20 × 0.12 × 0.07

Data collection	
Diffractometer	Bruker D8 Venture
No. of measured, independent and observed [*I* > 2σ(*I*)] reflections	53961, 4064, 3720
*R*_int_	0.050
(sin θ/λ)_max_ (Å^−1^)	0.719

Refinement	
*R*[*F*^2^ > 2σ(*F*^2^)], *wR*(*F*^2^), *S*	0.043, 0.137, 1.05
No. of reflections	4064
No. of parameters	172
H-atom treatment	H-atom parameters constrained
Δρ_max_, Δρ_min_ (e Å^−3^)	0.37, −0.28

Computer programs: *APEX4* and *SAINT* (Bruker, 2019 ▸), *SHELXS2018/2* (Sheldrick, 2015*a* ▸), *SHELXL2018/3* (Sheldrick, 2015*b* ▸), *PLATON* (Spek, 2020 ▸) and *publCIF* (Westrip, 2010 ▸).

## full crystallographic data

### 2-Oxo-2H-chromen-7-yl tert-butylacetate Crystal data

**Table:**

C_15_H_16_O_4_	*Z* = 2
*M**_r_* = 260.28	*F*(000) = 276
Triclinic, *P*1	*D*_x_ = 1.316 Mg m^−^^3^
Hall symbol: -P 1	Melting point: 368 K
*a* = 6.1599 (9) Å	Mo *K*α radiation, λ = 0.71073 Å
*b* = 7.2029 (11) Å	Cell parameters from 4066 reflections
*c* = 15.202 (2) Å	θ = 2.9–30.8°
α = 98.765 (5)°	µ = 0.10 mm^−^^1^
β = 99.335 (5)°	*T* = 100 K
γ = 91.228 (5)°	Prism, yellow
*V* = 657.05 (17) Å^3^	0.20 × 0.12 × 0.07 mm

### 2-Oxo-2H-chromen-7-yl tert-butylacetate Data collection

**Table:**

Bruker D8 Venture diffractometer	3720 reflections with *I* > 2σ(*I*)
Radiation source: fine-focus sealed tube	*R*_int_ = 0.050
Mirror monochromator	θ_max_ = 30.8°, θ_min_ = 2.9°
φ and ω scan	*h* = −8→8
53961 measured reflections	*k* = −10→10
4064 independent reflections	*l* = −21→21

### 2-Oxo-2H-chromen-7-yl tert-butylacetate Refinement

**Table:**

Refinement on *F*^2^	Primary atom site location: structure-invariant direct methods
Least-squares matrix: full	Secondary atom site location: difference Fourier map
*R*[*F*^2^ > 2σ(*F*^2^)] = 0.043	Hydrogen site location: inferred from neighbouring sites
*wR*(*F*^2^) = 0.137	H-atom parameters constrained
*S* = 1.05	*w* = 1/[σ^2^(*F*_o_^2^) + (0.0778*P*)^2^ + 0.2223*P*] where *P* = (*F*_o_^2^ + 2*F*_c_^2^)/3
4064 reflections	(Δ/σ)_max_ < 0.001
172 parameters	Δρ_max_ = 0.37 e Å^−^^3^
0 restraints	Δρ_min_ = −0.28 e Å^−^^3^

### 2-Oxo-2H-chromen-7-yl tert-butylacetate Special details

**Table:**

Geometry. All esds (except the esd in the dihedral angle between two l.s. planes) are estimated using the full covariance matrix. The cell esds are taken into account individually in the estimation of esds in distances, angles and torsion angles; correlations between esds in cell parameters are only used when they are defined by crystal symmetry. An approximate (isotropic) treatment of cell esds is used for estimating esds involving l.s. planes.

### 2-Oxo-2H-chromen-7-yl tert-butylacetate Fractional atomic coordinates and isotropic or equivalent isotropic displacement parameters (Å^2^)

**Table:**

	*x*	*y*	*z*	*U*_iso_*/*U*_eq_	
O3	0.53573 (12)	0.19418 (10)	0.29426 (5)	0.02062 (16)	
O1	0.88247 (11)	0.15470 (10)	0.59464 (5)	0.01913 (16)	
O4	0.84148 (12)	0.38219 (10)	0.30532 (5)	0.02129 (16)	
O2	1.04542 (14)	0.10787 (12)	0.72944 (5)	0.02755 (18)	
C9	0.51037 (15)	0.26016 (12)	0.56924 (6)	0.01750 (18)	
C6	0.53813 (16)	0.22422 (13)	0.38729 (6)	0.01796 (18)	
C5	0.34391 (15)	0.28555 (13)	0.41609 (7)	0.01937 (18)	
H5	0.222778	0.314675	0.374030	0.023*	
C4	0.33122 (15)	0.30309 (13)	0.50706 (7)	0.01946 (19)	
H4	0.200134	0.344549	0.527611	0.023*	
C8	0.70128 (15)	0.20004 (13)	0.53717 (6)	0.01673 (17)	
C7	0.71880 (15)	0.18000 (13)	0.44623 (6)	0.01781 (18)	
H7	0.849197	0.137745	0.425327	0.021*	
C10	0.70385 (15)	0.27069 (13)	0.26024 (6)	0.01775 (18)	
C1	0.88143 (17)	0.15948 (14)	0.68557 (6)	0.02053 (19)	
C3	0.50766 (17)	0.27137 (14)	0.66442 (7)	0.02070 (19)	
H3	0.380241	0.311803	0.688282	0.025*	
C2	0.68492 (18)	0.22481 (14)	0.71993 (7)	0.0225 (2)	
H2	0.681287	0.234916	0.782707	0.027*	
C11	0.68111 (16)	0.19153 (14)	0.16148 (6)	0.01956 (18)	
H11A	0.522872	0.186209	0.134991	0.023*	
H11B	0.728920	0.060533	0.156411	0.023*	
C12	0.80830 (16)	0.29648 (14)	0.10383 (6)	0.02054 (19)	
C13	1.05795 (18)	0.29276 (19)	0.13479 (8)	0.0305 (2)	
H13A	1.135176	0.360602	0.096951	0.046*	
H13B	1.096000	0.353175	0.197906	0.046*	
H13C	1.102008	0.162119	0.129266	0.046*	
C15	0.7374 (2)	0.50003 (16)	0.10722 (7)	0.0284 (2)	
H15A	0.820076	0.565015	0.070105	0.043*	
H15B	0.579442	0.500204	0.083962	0.043*	
H15C	0.767411	0.564855	0.169777	0.043*	
C14	0.74935 (19)	0.19270 (17)	0.00628 (7)	0.0277 (2)	
H14A	0.827400	0.255304	−0.033149	0.041*	
H14B	0.792917	0.062204	0.003871	0.041*	
H14C	0.590176	0.194470	−0.014024	0.041*	

### 2-Oxo-2H-chromen-7-yl tert-butylacetate Atomic displacement parameters (Å^2^)

**Table:**

	*U*^11^	*U*^22^	*U*^33^	*U*^12^	*U*^13^	*U*^23^
O3	0.0191 (3)	0.0257 (3)	0.0169 (3)	−0.0023 (3)	0.0020 (2)	0.0045 (3)
O1	0.0186 (3)	0.0219 (3)	0.0171 (3)	0.0041 (2)	0.0023 (2)	0.0040 (2)
O4	0.0204 (3)	0.0241 (3)	0.0187 (3)	−0.0014 (3)	0.0019 (2)	0.0032 (3)
O2	0.0274 (4)	0.0334 (4)	0.0216 (4)	0.0057 (3)	−0.0004 (3)	0.0074 (3)
C9	0.0181 (4)	0.0150 (4)	0.0201 (4)	0.0005 (3)	0.0050 (3)	0.0028 (3)
C6	0.0184 (4)	0.0182 (4)	0.0177 (4)	−0.0005 (3)	0.0030 (3)	0.0041 (3)
C5	0.0156 (4)	0.0199 (4)	0.0229 (4)	0.0003 (3)	0.0018 (3)	0.0058 (3)
C4	0.0163 (4)	0.0188 (4)	0.0244 (4)	0.0015 (3)	0.0057 (3)	0.0042 (3)
C8	0.0167 (4)	0.0155 (4)	0.0181 (4)	0.0014 (3)	0.0026 (3)	0.0034 (3)
C7	0.0175 (4)	0.0186 (4)	0.0180 (4)	0.0023 (3)	0.0041 (3)	0.0035 (3)
C10	0.0176 (4)	0.0189 (4)	0.0176 (4)	0.0031 (3)	0.0024 (3)	0.0054 (3)
C1	0.0244 (5)	0.0197 (4)	0.0173 (4)	0.0006 (3)	0.0024 (3)	0.0037 (3)
C3	0.0222 (4)	0.0195 (4)	0.0214 (4)	0.0010 (3)	0.0078 (3)	0.0019 (3)
C2	0.0274 (5)	0.0232 (4)	0.0174 (4)	0.0008 (4)	0.0060 (3)	0.0021 (3)
C11	0.0197 (4)	0.0213 (4)	0.0171 (4)	0.0010 (3)	0.0020 (3)	0.0029 (3)
C12	0.0202 (4)	0.0258 (4)	0.0156 (4)	0.0012 (3)	0.0030 (3)	0.0032 (3)
C13	0.0198 (5)	0.0468 (7)	0.0245 (5)	−0.0003 (4)	0.0048 (4)	0.0032 (4)
C15	0.0382 (6)	0.0250 (5)	0.0223 (5)	0.0003 (4)	0.0024 (4)	0.0075 (4)
C14	0.0294 (5)	0.0354 (6)	0.0172 (4)	0.0008 (4)	0.0040 (4)	0.0011 (4)

### 2-Oxo-2H-chromen-7-yl tert-butylacetate Geometric parameters (Å, º)

**Table:**

O3—C10	1.3710 (11)	C3—C2	1.3490 (14)
O3—C6	1.3953 (11)	C3—H3	0.9500
O1—C1	1.3785 (11)	C2—H2	0.9500
O1—C8	1.3791 (11)	C11—C12	1.5339 (14)
O4—C10	1.2033 (12)	C11—H11A	0.9900
O2—C1	1.2151 (12)	C11—H11B	0.9900
C9—C8	1.3970 (13)	C12—C15	1.5344 (15)
C9—C4	1.4044 (13)	C12—C13	1.5355 (15)
C9—C3	1.4396 (13)	C12—C14	1.5380 (14)
C6—C7	1.3862 (13)	C13—H13A	0.9800
C6—C5	1.3971 (13)	C13—H13B	0.9800
C5—C4	1.3845 (13)	C13—H13C	0.9800
C5—H5	0.9500	C15—H15A	0.9800
C4—H4	0.9500	C15—H15B	0.9800
C8—C7	1.3900 (13)	C15—H15C	0.9800
C7—H7	0.9500	C14—H14A	0.9800
C10—C11	1.5054 (13)	C14—H14B	0.9800
C1—C2	1.4540 (14)	C14—H14C	0.9800
			
C10—O3—C6	119.43 (7)	C1—C2—H2	119.3
C1—O1—C8	121.85 (8)	C10—C11—C12	117.18 (8)
C8—C9—C4	118.47 (9)	C10—C11—H11A	108.0
C8—C9—C3	117.80 (9)	C12—C11—H11A	108.0
C4—C9—C3	123.72 (9)	C10—C11—H11B	108.0
C7—C6—O3	120.83 (8)	C12—C11—H11B	108.0
C7—C6—C5	122.58 (9)	H11A—C11—H11B	107.2
O3—C6—C5	116.39 (8)	C11—C12—C15	110.51 (8)
C4—C5—C6	118.78 (9)	C11—C12—C13	111.12 (8)
C4—C5—H5	120.6	C15—C12—C13	110.36 (9)
C6—C5—H5	120.6	C11—C12—C14	106.66 (8)
C5—C4—C9	120.60 (9)	C15—C12—C14	109.16 (8)
C5—C4—H4	119.7	C13—C12—C14	108.94 (8)
C9—C4—H4	119.7	C12—C13—H13A	109.5
O1—C8—C7	116.34 (8)	C12—C13—H13B	109.5
O1—C8—C9	121.32 (8)	H13A—C13—H13B	109.5
C7—C8—C9	122.33 (9)	C12—C13—H13C	109.5
C6—C7—C8	117.24 (8)	H13A—C13—H13C	109.5
C6—C7—H7	121.4	H13B—C13—H13C	109.5
C8—C7—H7	121.4	C12—C15—H15A	109.5
O4—C10—O3	123.07 (9)	C12—C15—H15B	109.5
O4—C10—C11	128.49 (9)	H15A—C15—H15B	109.5
O3—C10—C11	108.45 (8)	C12—C15—H15C	109.5
O2—C1—O1	116.63 (9)	H15A—C15—H15C	109.5
O2—C1—C2	126.14 (9)	H15B—C15—H15C	109.5
O1—C1—C2	117.23 (9)	C12—C14—H14A	109.5
C2—C3—C9	120.42 (9)	C12—C14—H14B	109.5
C2—C3—H3	119.8	H14A—C14—H14B	109.5
C9—C3—H3	119.8	C12—C14—H14C	109.5
C3—C2—C1	121.30 (9)	H14A—C14—H14C	109.5
C3—C2—H2	119.3	H14B—C14—H14C	109.5

### 2-Oxo-2H-chromen-7-yl tert-butylacetate Hydrogen-bond geometry (Å, º)

**Table:**

*D*—H···*A*	*D*—H	H···*A*	*D*···*A*	*D*—H···*A*
C5—H5···O4^i^	0.95	2.50	3.4144 (13)	161
C11—H11*B*···O2^ii^	0.99	2.52	3.2523 (13)	131
C13—H13*B*···O4	0.98	2.43	3.0924 (14)	124

Symmetry codes: (i) *x*−1, *y*, *z*; (ii) −*x*+2, −*y*, −*z*+1.
